# An Analytical Framework for Runtime of a Class of Continuous Evolutionary Algorithms

**DOI:** 10.1155/2015/485215

**Published:** 2015-08-12

**Authors:** Yushan Zhang, Guiwu Hu

**Affiliations:** School of Mathematics and Statistics, Guangdong University of Finance and Economics, Guangzhou 510320, China

## Abstract

Although there have been many studies on the runtime of evolutionary algorithms in discrete optimization, relatively few theoretical results have been proposed on continuous optimization, such as evolutionary programming (EP). This paper proposes an analysis of the runtime of two EP algorithms based on Gaussian and Cauchy mutations, using an absorbing Markov chain. Given a constant variation, we calculate the runtime upper bound of special Gaussian mutation EP and Cauchy mutation EP. Our analysis reveals that the upper bounds are impacted by individual number, problem dimension number *n*, searching range, and the Lebesgue measure of the optimal neighborhood. Furthermore, we provide conditions whereby the average runtime of the considered EP can be no more than a polynomial of *n*. The condition is that the Lebesgue measure of the optimal neighborhood is larger than a combinatorial calculation of an exponential and the given polynomial of *n*.

## 1. Introduction

The running time to optimum is a key factor in determining the success of an evolutionary programming (EP) approach. Ideally, an implementation of an EP approach should run for a sufficient number of generations when the probability of achieving an optimum is greater than some desired value. However, few results on the running time of EP approaches can be found in the current literature.

As a technique of finite state machine, EP was first proposed for continuous optimization [1]. It has since been widely adopted as a powerful optimizing framework for solving continuous optimization problems [2–4]. More recently, EP research has mainly concentrated on the function of its parameters and its improvement by adaptive strategies. As a result, several EP variants have been proposed, distinguishing from each other mainly with different mutation schemes based on different probability distributions.

Arguably, the first EP algorithm that was widely considered successful was the one with Gauss mutation that has been termed as* classical evolutionary programming* (CEP) [2]. CEP has been intensively analyzed by Fogel [3, 4], Bäck and Schwefel [5], and Schwefel [5, 6]. Subsequently, Yao et al. proposed a* fast evolutionary programming algorithm* (FEP) with a new mutation scheme based on the Cauchy distribution [7]. Computational experiments showed that FEP is superior to CEP when tackling the optimization problems of multimodal and dispersed peak functions. Another EP variant [8] was later proposed by using the Lévy distribution based mutation, which we call* Lévy evolutionary programming* (LEP) in this paper. Empirical analyses show that, overall, LEP exceeds CEP and FEP when solving the benchmark problems of multimodal and highly dispersed peak functions.

CEP [2], FEP [7], and LEP [8] can be considered as classical evolutionary programming algorithms. Several modified types of EP have since been designed based on these three basic approaches [2, 7, 8].

The performances of EP approaches such as CEP, FEP, and LEP have oftentimes been verified experimentally rather than theoretically. The theoretical foundations of EP have been an open problem ever since it was first put forward [1, 2]. In particular, Bäck and Schwefel [5] and Schwefel [6] suggested the convergence analysis of EP algorithms as a research topic in their surveys of evolutionary algorithms. Fogel [2–4] presented an initial proof of EP convergence on the basis of the discrete solution space. Rudolph [9–11] then showed that CEP and FEP can converge with an arbitrary initialization; this result is more general since it applies to a continuous solution space.

Previous convergence studies only considered whether an EP algorithm is able to find an optimum within infinite iteration but did not mention the speed of convergence, that is, lacking running time analysis. To date, running time analyses have mainly focused on Boolean individual EAs like (1 + 1)EA [12], (*N* + *N*)EA [13], multiobjective EA [14–16], and so forth. Alternative theoretical measures for evaluating the running time of Boolean-individual EA approaches have also been proposed [17, 18]. Recently, the impact of particular components of EA on runtime has also been studied, on mutation, selection [19], and population size [18, 20, 21]. In addition to these studies on EAs solving Boolean functions, some results of runtime analysis have been obtained on some combinatorial optimization problems [22–24]. Lehre and Yao [25] completed the runtime analysis of the (1 + 1)EA on computing unique input output sequences. Zhou et al. presented a series of EA analysis results for discrete optimization like the minimum label spanning tree problem [26], the multiprocessor scheduling problem [27], and the maximum cut problem [28]. Other proposed studies are on the topics of tight bounds on the running time of EA and randomized search heuristic [29–31].

As summarized above, the majority of runtime analyses are limited to discrete search space; analyses for continuous search space require a more sophisticated modeling and remain relatively few, which is unsatisfactory from a theoretical point of view. Jägersküpper conducted a rigorous runtime analysis on (1 + 1)ES, (1 + *λ*)ES minimizing the Sphere function [32, 33]. Agapie et al. modeled the continuous EA as a renewal process under some strong assumption and analyzed the first hitting time of (1 + 1)EA on inclined plane and Sphere function [34, 35]. Chen et al. [36] proposed general drift conditions to estimate the upper bound of the first hitting time for EAs to find *ϵ*-approximation solutions. Inspired by the studies above, especially the estimating approach in [17] for discrete situation, this paper presents an analytical framework for the running time of evolutionary programming. We also discuss whether the running time of CEP and FEP is influenced by individual number, problem dimension number, searching range, and the Lebesgue measure of the optimal neighborhood in the target problem. Furthermore, we give the approximate conditions under which the EP can converge in a timespan less than a polynomial of *n*.

## 2. EP Algorithms and the Associated Markov Chain

### 2.1. Introduction to EP Algorithms

This section introduces the two EP algorithms CEP [2] and FEP [7] that are studied in this paper. The skeleton of EP algorithms analyzed in this paper is given in Algorithm 1; the sole difference among CEP, FEP, and LEP lies in their treatments of Step  (3).

**Algorithm 1 (framework of EP algorithms).**

1. Generate individuals randomly.
2. Calculate the fitness of each individual.
3. Generate offspring by mutation.
4. Evaluate the offspring.
5. Select individuals by a tournament rule.
6. If the terminal condition is satisfied, output the best-so-far solution found and exit; otherwise, go back to Step  (3).

In Step (1), the generated *k* individuals are denoted by a couple of real vectors **v**_*i*_ = (**x**_*i*_, **σ**_*i*_), *i* = 1,2,…, *k*, where the elements **x**_*i*_ = (*x*_*i*1_, *x*_*i*2_,…, *x*_*in*_) are the variables to be optimized, and the elements **σ**_*i*_ = (*σ*_*i*1_, *σ*_*i*2_,…, *σ*_*in*_) are* variation variables* that affect the generation of offspring. We set the iteration number *t* = 0 and the initial *σ*_*ij*_ ≤ 2  (*j* = 1,…, *n*) as proposed in [8]. The fitness values in Steps  (2) and (4) are calculated according to the objective function of the target problem.

In Step  (3), a single ${\bar{v}}_{i}=({\bar{x}}_{i},{\bar{\sigma }}_{i})$ is generated for each individual **v**_*i*_, where *i* = 1,…, *k*. For *j* = 1,…, *n*,

(1) $$\begin{array}{c} {\bar{\sigma }}_{ij}=V\left(\;\sigma_{ij}\;\right), \end{array}$$

where *V*(*σ*_*ij*_) denotes a renewing function of variation variable *σ*_*ij*_. The renewing function ${\bar{\sigma }}_{ij}=V(\sigma_{ij})$ may have various forms, and a representative implementation of it can be found in [7]. However, for ease of theoretical analysis, we will consider a kind of simple renewing function, that is, constant function, in our case studies (see Section 4):

(2) $$\begin{array}{c} V\left(\;\sigma_{ij}\;\right)=\sigma >0. \end{array}$$

The three EP algorithms differ in how ${\bar{x}}_{i}$ is derived, which can be explained as follows (in this paper, we only consider CEP and FEP):

(3) CEP:  x¯ij=xij+σ¯ijNj0,1,

(4) FEP:  x¯ij=xij+σ¯ijδj,

where *N*_*j*_(0,1) is a newly generated random variable by Gaussian distribution for each *j* and *δ*_*j*_ is a Cauchy random number produced anew for *j*th dimension, whose density function is

(5) $$\begin{array}{c} C_{\phi =1}\left(\;y\;\right)=\pi^{-1}\cdot {\left(\;1+y^{2}\;\right)}^{-1}\;-\infty <y<+\infty . \end{array}$$

In Step  (5), for each individual in the set of all parents and offsprings, *q* different* opponent* individuals are uniformly and randomly selected to be compared. If the selected individual's fitness value is more than the opponent's, the individual obtains a “win.” The top *k* individuals with the most “wins” are selected to be the parents in the next iteration, breaking ties by greater fitness.

### 2.2. Target Problem of EP Algorithms

Without loss of generality, we assume that the EP approaches analyzed in this study aim to solve a minimization problem in a continuous search space, defined as follows.

**Definition 2 (minimization problem).**

Let **S**⊆**R** ^*n*^ be a finite subspace of the *n*-dimensional real domain **R** ^*n*^, and let *f* : **S** → **R** be an *n*-dimensional real function. A minimization problem, denoted by the 2-tuple (**S**, *f*), is to find an *n*-dimensional vector **x**_min_ ∈ **S** such that, ∀**x** ∈ **S**, *f*(**x**_min_) ≤ *f*(**x**).

Without loss of generality, we can assume that **S** = ∏_*i*=1_^*n*^[−*b*_*i*_, *b*_*i*_] where *b*_*i*_ = *b* > 0. The function *f* : **S** → **R** is called the* objective function* of the minimization problem. We do not require *f* to be continuous, but it must be bounded. Furthermore, we only consider the unconstrained minimization.

The following properties are assumed, and we will make use of them in our analyses:

(1) The subset of optimal solutions in **S** is nonempty.
(2) Let *f* ^*∗*^ = min{*f*(*x*)∣*x* ∈ **S**} be the fitness value of an optimal solution, and let **S** ^*∗*^(*ɛ*) = {*x* ∈ **S**∣*f*(*x*) < *f* ^*∗*^ + *ɛ*} be the optimal epsilon neighborhood. Every element of **S** ^*∗*^(*ɛ*) is an optimal solution of the minimization problem.
(3) ∀*ɛ* > 0, *m*(**S** ^*∗*^(*ɛ*)) > 0, where we denote the Lebesgue measure of **S** ^*∗*^(*ɛ*) as *m*(**S** ^*∗*^(*ɛ*)).

The first assumption describes the existence of optimal solutions to the problem. The second assumption presents a rigorous definition of optimal solution for continuous minimization optimization problems. The third assumption indicates that there are always solutions whose objective values are distributed continuously and arbitrarily close to the optimal, which makes the minimization problem solvable.

### 2.3. Markov Chain Associated with EP Algorithms

Our running time analyses are based on representing the EP algorithms as Markov chain. In this section, we explain the terminologies and notations used throughout the rest of this study.

**Definition 3 (stochastic process of EP).**

The stochastic process of an evolutionary programming algorithm EP is denoted by {*ξ*_*t*_^EP^}_*t*=0_^+*∞*^; *ξ*_*t*_^EP^ = (**v**_1_^(*t*)^, **v**_2_^(*t*)^,…, **v**_*k*_^(*t*)^), where **v**_*i*_^(*t*)^ = (**x**_*i*_^(*t*)^, **σ**_*i*_^(*t*)^) is the *i*th individual at the *t*th iteration.

The stochastic status *ξ*_*t*_^EP^ represents the individuals of the *t*th iteration population for the algorithm EP. All stochastic processes examined in this paper are discrete-time; that is, *t* ∈ **Z** ^+^.

**Definition 4 (status space).**

The status space of EP is *Ω*_EP_ = {(**v**_1_, **v**_2_,…, **v**_*k*_)∣**v**_*i*_ = (**x**_*i*_, **σ**_*i*_), **x**_*i*_ ∈ **S**, *σ*_*ij*_ ≤ 2, *j* = 1,…, *n*}.

*Ω*_EP_ is the set of all possible population statuses for EP. Intuitively, each element of *Ω*_EP_ is associated with a possible population in the implementation of EP. Let **x** ^*∗*^ ∈ **S** ^*∗*^(*ɛ*) be an optimal solution. We define the optimal status space as follows.

**Definition 5 (optimal status space).**

The optimal status space of EP is the subset *Ω*_EP_^*∗*^(*ɛ*)⊆*Ω*_EP_ such that, ∀**u** ^*∗*^ ∈ *Ω*_EP_^*∗*^(*ɛ*), ∃(**x** ^*∗*^, **σ**) ∈ **u** ^*∗*^, where **σ** = (*σ*, *σ*,…, *σ*) and *σ* > 0.

Hence, all members of *Ω*_EP_^*∗*^(*ɛ*) contain at least one optimal solution represented by individual **x** ^*∗*^.

**Definition 6 (Markov chain).**

A stochastic process {*ξ*_*t*_}_*t*=0_^+*∞*^ with status space *Ω* is a Markov chain if $\forall \tilde{\Omega }\subseteq \Omega$, $P\{\xi_{t+1}\in \tilde{\Omega }|\xi_{0},\ldots ,\xi_{t}\}=P\{\xi_{t+1}\in \tilde{\Omega }|\xi_{t}\}$.

**Lemma 7 .**

The stochastic process {*ξ*_*t*_^*EP*^}_*t*=0_^+*∞*^ of EP is a Markov chain.

**Proof**

The proof is given in the appendix.

We now show that the stochastic process {*ξ*_*t*_^EP^}_*t*=0_^+*∞*^ of EP is an absorbing Markov chain, defined as follows.

**Definition 8 (absorbing Markov chain to optimal status space).**

A Markov chain {*ξ*_*t*_}_*t*=0_^+*∞*^ is an absorbing Markov chain to *Ω* ^*∗*^ if ∃*Ω* ^*∗*^ ⊂ *Ω*, such that *P*{*ξ*_*t*+1_ ∉ *Ω* ^*∗*^∣*ξ*_*t*_ ∈ *Ω* ^*∗*^} = 0 for *t* = 0,1,….

**Lemma 9 .**

The stochastic process {*ξ*_*t*_^*EP*^}_*t*=0_^+*∞*^ of EP is an absorbing Markov chain to *Ω*_*EP*_^*∗*^(*ɛ*).

**Proof**

The proof is given in the appendix.

This property implies that once an EP algorithm attains an optimal state, it will never leave optimality.

## 3. General Runtime Analysis Framework for EP Algorithms

The analysis of EP algorithms has usually been approximated using a simpler measure known as the first hitting time [13, 17], which is employed in this study.

**Definition 10 (first hitting time of EP).**

*μ*_EP_ is the first hitting time of EP if *μ*_EP_ = min{*t* ≥ 0 : *ξ*_*t*_^EP^ ∈ *Ω*_EP_^*∗*^(*ɛ*)}.

If an EP algorithm is modeled as an absorbing Markov chain, the running time of the EP can be measured by its first hitting time *μ*_EP_. We denote its expected value by *Eμ*_EP_ which can be calculated by Theorem 11.

Let *λ*_*t*_^EP^ = *P*{*ξ*_*t*_^EP^ ∈ *Ω*_EP_^*∗*^(*ɛ*)} = *P*{*μ*_EP_ ≤ *t*} be the probability that EP has attained an optimal state by time *t*.

**Theorem 11 .**

If lim_*t*→+*∞*_⁡*λ*_*t*_^*EP*^ = 1, the expected first hitting time *Eμ*_*EP*_ = ∑_*i*=0_^+*∞*^(1 − *λ*_*i*_^*EP*^).

**Corollary 12 .**

The expected first hitting time can also be expressed as *Eμ*_*EP*_ = (1 − *λ*_0_^*EP*^)∑_*t*=0_^+*∞*^∏_*i*=1_^*t*^(1 − *P*{*ξ*_*i*_^*EP*^ ∈ *Ω*_*EP*_^*∗*^(*ɛ*)∣*ξ*_*i*−1_^*EP*^ ∉ *Ω*_*EP*_^*∗*^(*ɛ*)}).

**Proof**

The proof is given in the appendix.

The conclusions of Theorem 11 and Corollary 12 are a direct approach to calculating the first hitting time of EP. However, Corollary 12 is more practical than Theorem 11 for this purpose since *p*_*i*_ = *P*{*ξ*_*i*_^EP^ ∈ *Ω*_EP_^*∗*^(*ɛ*)∣*ξ*_*i*−1_^EP^ ∉ *Ω*_EP_^*∗*^(*ɛ*)}, which is the probability that the process first finds an optimal solution at time *i*, is dependent on the mutation and selection techniques of the EP. Hence, the framework of Corollary 12 is similar to the one in [17]. However, the estimating method [17] is based on a probability *q*_*i*_ in discrete optimization *q*_*i*_ = ∑_*y*∉*Ω*_EP_^*∗*^(*ɛ*)_ *P*{*ξ*_*i*_^EP^ ∈ *Ω*_EP_^*∗*^(*ɛ*)∣*ξ*_*i*−1_^EP^ = *y*}*P*{*ξ*_*i*−1_^EP^ = *y*}. *p*_*i*_ is a probability for continuous status while *q*_*i*_ is for the discrete one. In general, the exact value of *p*_*i*_ is difficult to calculate. Alternatively, first hitting time can also be analyzed in terms of an upper and a lower bound of *p*_*i*_, as shown by the following Corollaries 13 and 15.

**Corollary 13 .**

If *α*_*t*_ ≤ *P*{*ξ*_*t*_^*EP*^ ∈ *Ω*_*EP*_^*∗*^(*ɛ*)∣*ξ*_*t*−1_^*EP*^ ∉ *Ω*_*EP*_^*∗*^(*ɛ*)} ≤ *β*_*t*_,

(1) ∑_*t*=0_^+*∞*^[(1 − *λ*_0_^*EP*^)∏_*i*=1_^*t*^(1 − *β*_*i*_)] ≤ *Eμ*_*EP*_ ≤ ∑_*t*=0_^+*∞*^[(1 − *λ*_0_^*EP*^)∏_*i*=1_^*t*^(1 − *α*_*i*_)] where 0 < *α*_*t*_ and *β*_*t*_ < 1;
(2) *β* ^−1^(1 − *λ*_0_^*EP*^) ≤ *Eμ*_*EP*_ ≤ *α* ^−1^(1 − *λ*_0_^*EP*^) when *α*_*t*_ = *α* and *β*_*t*_ = *β*.

**Proof**

The proof is given in the appendix.

Corollary 13 indicates that *Eμ*_EP_ can be studied by the lower bound and upper bound of *P*{*ξ*_*t*_^EP^ ∈ *Ω*_EP_^*∗*^(*ɛ*)∣*ξ*_*t*−1_^EP^ ∉ *Ω*_EP_^*∗*^(*ɛ*)}. Therefore, the theorem and corollaries introduce a first-hitting-time framework for the running time analysis of EP. The running times of EP based on Gaussian and Cauchy mutation are studied following the framework.

## 4. Runtime Upper Bounds of CEP and FEP

In this section, we use the framework proposed in Section 3 to study the running time of EP based on Gaussian and Cauchy mutations, that is, CEP and FEP. Moreover, our running time analysis aims at a class of EP with constant variation, as shown in (2).

### 4.1. Mean Running Time of CEP

CEP [2] is a classical EP, from which several continuous evolutionary algorithms are derived. This subsection mainly focuses on the running time of CEP proposed by Fogel [3], Schwefel [6], and Schwefel [5, 6]. The mutation of CEP is based on the standard normal distribution indicated by (3).

According to the running time analysis framework, the probability *p*_*t*_ = *P*{*ξ*_*t*_^*C*^ ∈ *Ω*_EP_^*∗*^(*ɛ*)∣*ξ*_*t*−1_^*C*^ ∉ *Ω*_EP_^*∗*^(*ɛ*)} for CEP is a crucial factor, and its property is introduced by the following.

**Theorem 14 .**

Let the stochastic process of CEP be denoted by {*ξ*_*t*_^*C*^}_*t*=0_^+*∞*^. Let *k* be the population size of CEP with solution space **S** = ∏_*i*=1_^*n*^[−*b*_*i*_, *b*_*i*_] where *b*_*i*_ = *b* > 0. Then ∀*ɛ* > 0, one has the following:

(1) For fixed individual $\left(x,\bar{\sigma }\right)$, $P\{\bar{x}\in S^{*}(ɛ)\}\geq (m\left(S^{*}\left(ɛ\right)\right)/{\left(\sqrt{2\pi }\right)}^{n})(\prod_{j=1}^{n}\left(1/\sigma \right))exp\{-\sum_{j=1}^{n}\left(2b^{2}/\sigma^{2}\right)\}$ where the renewing function *V*(*σ*_*ij*_) = *σ*.
(2) The right part of the inequality of conclusion (1) is maximum when *σ* = 2*b*.
(3) $P\{\xi_{t}^{C}\in \Omega_{EP}^{*}(ɛ)|\xi_{t-1}^{C}\notin \Omega_{EP}^{*}(ɛ)\}\geq 1-{\left(1-m(S^{*}(ɛ))/{\left(4\sqrt{e}\pi b\right)}^{n}\right)}^{k}$ if *σ* = 2*b*.

**Proof**

The proof is given in the appendix.

In Theorem 14, Part (1) indicates a lower bound of the probability that an individual **x** can be renewed to be $\bar{x}$ in the optimal neighborhood **S** ^*∗*^(*ɛ*). As a result, the lower bound is a function of variation *σ*. Part (2) presents the lower bound that Part (1) will be tightest if *σ* = 2*b*. By this, the key factor of Corollary 13 *P*{*ξ*_*t*_^*C*^ ∈ *Ω*_EP_^*∗*^(*ɛ*)∣*ξ*_*t*−1_^*C*^ ∉ *Ω*_EP_^*∗*^(*ɛ*)} may have a tight lower bound shown by Part (3).

Theorem 14 indicates that a larger *m*(**S** ^*∗*^(*ɛ*)) leads to faster convergence for CEP since *P*{*ξ*_*t*_^*C*^ ∈ *Ω*_EP_^*∗*^(*ɛ*)∣*ξ*_*t*−1_^*C*^ ∉ *Ω*_EP_^*∗*^(*ɛ*)} becomes larger when *m*(**S** ^*∗*^(*ɛ*)) is larger. Moreover, Theorem 14 produces the lower bound of *P*{*ξ*_*t*_^*C*^ ∈ *Ω*_EP_^*∗*^(*ɛ*)∣*ξ*_*t*−1_^*C*^ ∉ *Ω*_EP_^*∗*^(*ɛ*)}, with which the first-hitting time of CEP can be estimated following Corollary 13.  Corollary 15 indicates the convergence capacity and running time upper bound of CEP.

**Corollary 15 .**

Supposed conditions of Theorem 14 are satisfied:

(1) lim_*t*→+*∞*_⁡*λ*_*t*_^*C*^ = 1  (*λ*_*t*_^*C*^ = *P*{*ξ*_*t*_^*C*^ ∈ *Ω*_*EP*_^*∗*^(*ɛ*)});
(2) ∀*ɛ* > 0,
  (6) $$\begin{array}{c} E\mu_{C}\leq \left(\;1-\lambda_{0}^{C}\;\right){\left(\;1-{\left(\;1-\frac{m\left(S^{*}\left(ɛ\right)\right)}{{\left(4\sqrt{e}\pi b\right)}^{n}}\;\right)}^{k}\;\right)}^{-1}. \end{array}$$

**Proof**

The proof is given in the appendix.

Corollary 15 shows that CEP converges globally and that the upper bound of CEP's running time decreases as the Lebesgue measure of the optimal *ɛ* neighborhood *m*(**S** ^*∗*^(*ɛ*)) of **S** ^*∗*^(*ɛ*) increases. A larger *m*(**S** ^*∗*^(*ɛ*)) translates to a larger optimal *ɛ* neighborhood ∀*ɛ* > 0, which allows the solution of CEP more easily. Moreover, an increase in problem dimension number can increase the upper bound, and enlarging the individual number will make the upper bound smaller.

According to (6), *Eμ*_*C*_ has a smaller upper bound when the population size *k* increases. Approximately,

(7) $$\begin{array}{c} E\mu_{C}\leq \left(\;1-\lambda_{0}^{C}\;\right)\frac{{\left(4\sqrt{e}\pi b\right)}^{n}}{km\left(S^{*}\left(ɛ\right)\right)} \end{array}$$

since ${\left(1-m\left(S^{*}\left(ɛ\right)\right)/{\left(4\sqrt{e}\pi b\right)}^{n}\right)}^{k}\approx 1-k(m(S^{*}(ɛ))/{\left(4\sqrt{e}\pi b\right)}^{n})$ when *k* is large. Furthermore, the running time of CEP is similar to an exponential order function of size *n*, if *m*(**S** ^*∗*^(*ɛ*)) ≥ *C*_0_ > 0 where *C*_0_ is a positive constant. Hence, the running time of CEP is nearly $O({\left(4\sqrt{e}\pi b\right)}^{n})$.

Moreover, we can give an approximate condition under which CEP can converge in time polynomial to *n* when $m(S^{*}(ɛ))\geq c_{C}({\left(4\sqrt{e}\pi b\right)}^{n}/P_{n})$, where *c*_*C*_ = (1 − *λ*_0_^*C*^)/*P*_*n*_ > 0.

Suppose (7) holds. Then $m(S^{*}(ɛ))\geq (1-\lambda_{0}^{C}){\left(4\sqrt{e}\pi b\right)}^{n}/P_{n}^{2}>0\Leftrightarrow E\mu_{C}\leq (1-\lambda_{0}^{C}){\left(4\sqrt{e}\pi b\right)}^{n}/P_{n}\cdot m(S^{*}(ɛ))\leq P_{n}.$ Thus, the running time of CEP can be polynomial to *n*, under the constraint that, ∀*ɛ* > 0, $m(S^{*}(ɛ))\geq (\left(1-\lambda_{0}^{C}\right)/k)\cdot ({\left(4\sqrt{e}\pi b\right)}^{n}/P_{n})$, where **S** ^*∗*^(*ɛ*) = {**x** ∈ **S**∣*f*(**x**) < *f* ^*∗*^ + *ɛ*}.

### 4.2. Mean Running Time of FEP

FEP was proposed by Yao et al. [7] as an improvement to CEP. The mutation for FEP is based on the Cauchy distribution indicated by (4). The property of the probability *p*_*t*_ = *P*{*ξ*_*t*_^*F*^ ∈ *Ω*_EP_^*∗*^(*ɛ*)∣*ξ*_*t*−1_^*F*^ ∉ *Ω*_EP_^*∗*^(*ɛ*)} is discussed by Theorem 16  (*t* = 0,1,…).

**Theorem 16 .**

Let {*ξ*_*t*_^*F*^}_*t*=0_^+*∞*^ be the stochastic process of FEP, and then one has the following:

(1) $P\{\bar{x}\in S^{*}(ɛ)\}\geq (m\left(S^{*}\left(ɛ\right)\right)/{\left(\pi \right)}^{n}){\left(\sigma +4b^{2}/\sigma \right)}^{-n}$.
(2) The right part of the inequality of conclusion (1) is maximum when *σ* = 2*b*.
(3) *P*{*ξ*_*t*_^*F*^ ∈ *Ω*_*EP*_^*∗*^(*ɛ*)∣*ξ*_*t*−1_^*F*^ ∉ *Ω*_*EP*_^*∗*^(*ɛ*)} ≥ 1 − (1 − *m*(**S** ^*∗*^(*ɛ*))/(4*πb*)^*n*^)^*k*^ if *σ* = 2*b*.

**Proof**

The proof is given in the appendix.

Similar to Theorem 14, FEP with *σ* = 2*b* may lead to a tight lower bound of the probability in Part (1) and Part (3). Theorem 16 indicates that *m*(**S** ^*∗*^(*ɛ*)) affects the convergence of FEP directly. A larger *m*(**S** ^*∗*^(*ɛ*)) allows FEP to more easily arrive at a status in the optimal state space (Definition 5). Conversely, a bigger *b* (the bounds of search space) increases the difficulty of the problem for FEP. Given this convergence time framework, Corollary 17 summarizes the convergence capacity and running time upper bound of FEP.

**Corollary 17 .**

Supposed conditions of Theorem 16 are satisfied:

(1) lim_*t*→+*∞*_⁡*λ*_*t*_^*F*^ = 1  (*λ*_*t*_^*F*^ = *P*{*ξ*_*t*_^*F*^ ∈ *Ω*_*EP*_^*∗*^(*ɛ*)});
(2) ∀*ɛ* > 0, *Eμ*_*F*_ ≤ (1 − *λ*_0_^*F*^)(1 − (1 − *m*(**S** ^*∗*^(*ɛ*))/(4*bπ*)^*n*^)^*k*^).

**Proof**

The proof is given in the appendix.

Corollary 17 proves the convergence of FEP and indicates that a larger *m*(**S** ^*∗*^(*ɛ*)) can make FEP converge faster. Using a similar analysis to that for CEP, a larger dimension number of problems *n* can increase the upper bound of FEP, but larger individual number *k* can lead to a smaller upper bound. Furthermore, [−*b*, *b*] is the maximum interval bound for each dimension of the variables to be optimized (Definition 2). As a result, the second conclusion of Corollary 17 also implies that a larger search space will increase the upper bound of *Eμ*_EP_.

If *k* becomes large enough, we have (1 − *m*(**S** ^*∗*^(*ɛ*))/(4*bπ*)^*n*^)^*k*^ ≈ 1 − *k*(*m*(**S** ^*∗*^(*ɛ*))/(4*bπ*)^*n*^) such that

(8) $$\begin{array}{c} E\mu_{F}\leq \frac{1-\lambda_{0}^{F}}{km\left(S^{*}\left(ɛ\right)\right)}{\left(\;4b\pi \;\right)}^{n}. \end{array}$$

Hence, the running time of FEP is nearly *O*((4*bπ*)^*n*^) when *m*(**S** ^*∗*^(*ɛ*)) is a constant greater than zero.

Moreover, we can give an approximate condition where FEP can converge in polynomial time *P*(*n*); that is, *m*(**S** ^*∗*^(*ɛ*))≥((1 − *λ*_0_^*F*^)/*k*)·((4*bπ*)^*n*^/*P*(*n*)), ∀*ɛ* > 0 when (8) is true. The analysis is similar to the one for CEP at the end of Section 4.1.

## 5. Conclusion

In this paper, we propose a running time framework to calculate the mean running time of EP. Based on this framework, the convergence and mean running time of CEP and FEP with constant variation are studied. We also obtain some results at the worst running time of the considered EP, although the results show that the upper bounds can be tighter if the variation *σ* = 2*b* where 2*b* is the length of the searching interval per dimension. It is shown that the individual number, problem dimension number, searching range, and the Lebesgue measure of the optimal neighborhood of the optimal solution have a direct impact on the bounds of the expected convergence times of the considered EP. Moreover, the larger the Lebesgue measure of the optimal neighborhood of the optimum, the lower the upper bound of the mean convergence time. In addition, the convergence time of the EP can be polynomial on average on the condition that the Lebesgue measure is greater than a value that is exponential to *b*.

However, it is possible to make an improvement on the running time framework and analysis given in this study. The deduction process in the proofs for Theorems 14 and 16 uses few properties of the distribution functions of the mutations, and so it is possible to tighten the results. By introducing more information on the specific mutation operations, more sound theoretical conclusions may be derivable. More importantly, unlike the rigorous conditions under which CEP and FEP can converge in polynomial time, the running time analysis of specific EP algorithms for real-world and constructive problems would have a more significant and practical impact. Future research could also focus on the runtime analysis of specific case studies of EP.

The proposed framework and results can be considered as a first step for analyzing the running time of evolutionary programming algorithms. We hope that our results can serve as a basis for further theoretical studies.

## Appendix

### Proof of the Lemmas, Theorems, and Corollaries

Proof of Lemma 7. Recall that *ξ*_*t*_^EP^ = (**v**_1_^(*t*)^, **v**_2_^(*t*)^,…, **v**_*k*_^(*t*)^) is a real vector and the status space *Ω*_EP_ of EP is continuous. Furthermore, *ξ*_*t*_^EP^ is only dependent on *ξ*_*t*−1_^EP^ for *t* = 0,1,…, as evidenced by Steps  (3)–(6) of the EP process. Hence, {*ξ*_*t*_^EP^}_*t*=0_^+*∞*^ is a Markov chain.

Proof of Lemma 9. 
*ξ*_*t*_^EP^ ∈ *Ω*_EP_^*∗*^(*ɛ*) when ∃(**x**_*i*_^*∗*(*t*)^, **σ**_*i*_^(*t*)^) ∈ *ξ*_*t*_^EP^ where **x**_*i*_^*∗*(*t*)^ is an optimal solution. Given Steps  (5)-(6) of EP, **x**_*i*_^*∗*(*t*)^ will have the most “wins” as well as the highest fitness, so **x**_*i*_^*∗*(*t*)^ will be selected for the next iteration with probability one if there are less than *k* optimal solutions in the tournament. **x**_*i*_^*∗*(*t*)^ might be lost but other optimal solutions will be selected if there are more than *k* optimal solutions in the parent and offspring. Thus, *P*{*ξ*_*t*+1_^EP^ ∉ *Ω*_EP_^*∗*^(*ɛ*)∣*ξ*_*t*_^EP^ ∈ *Ω*_EP_^*∗*^(*ɛ*)} = 0, and {*ξ*_*t*_^EP^}_*t*=0_^+*∞*^ is an absorbing Markov chain to *Ω*_EP_^*∗*^(*ɛ*).

Proof of Theorem 11. Observe that *λ*_*t*_^EP^ − *λ*_*t*−1_^EP^ = *P*{*μ*_EP_ ≤ *t*} − *P*{*μ*_EP_ ≤ *t* − 1} = *P*{*μ*_EP_ = *t*}. Therefore, we have

(A.1) EμEP∑t=1+∞t·λtEP−λt−1EP=limN→+∞⁡∑t=1Nt·λtEP−λt−1EP=limN→+∞⁡∑i=1NλNEP−λi−1EP=∑i=1+∞1−λi−1EP=∑i=0+∞1−λiEP.

Proof of Corollary 12. Let *p*_*i*_ = *P*{*ξ*_*i*_^EP^ ∈ *Ω*_EP_^*∗*^(*ɛ*)∣*ξ*_*i*−1_^EP^ ∉ *Ω*_EP_^*∗*^(*ɛ*)}. Based on the total probability equation, *λ*_*t*_^EP^ = (1 − *λ*_*t*−1_^EP^)*p*_*t*_ + *λ*_*t*−1_^EP^(1 − *p*_*t*_). Substituting it into Theorem 11, we have *Eμ*_EP_ = ∑_*t*=0_^+*∞*^[(1 − *λ*_0_^EP^)∏_*i*=1_^*t*^(1 − *p*_*i*_)].

Proof of Corollary 13. According to Corollary 12, *Eμ*_EP_ ≥ ∑_*t*=0_^+*∞*^[(1 − *λ*_0_^EP^)∏_*i*=1_^*t*^(1 − *β*_*i*_)]. Similarly, we have *Eμ*_EP_ ≤ ∑_*t*=0_^+*∞*^[(1 − *λ*_0_^EP^)∏_*i*=1_^*t*^(1 − *α*_*i*_)]. Thus, *β* ^−1^(1 − *λ*_0_^EP^) ≤ *Eμ*_EP_ ≤ *α* ^−1^(1 − *λ*_0_^EP^) when *α*_*t*_ = *α* and *β*_*t*_ = *β*.

Proof of Theorem 14. (1) For fixed individual $\left(x,\bar{\sigma }\right)$, $\bar{x}=x+u⊗\bar{\sigma }$, where $\bar{\sigma }=({\bar{\sigma }}_{1},{\bar{\sigma }}_{2},\ldots ,{\bar{\sigma }}_{n})$ is *n*-dimension variation variable and the stochastic disturbance **u** = (*u*_1_, *u*_2_,…, *u*_*n*_) follows *n*-dimensional standard Gaussian distribution. Let **z** = (*z*_1_, *z*_2_,…, *z*_*n*_); that is, $z=u⊗\bar{\sigma }=(u_{1}{\bar{\sigma }}_{1},u_{2}{\bar{\sigma }}_{2},\ldots ,u_{n}{\bar{\sigma }}_{n})$. According to (1) and (2), $z_{j}=\mu_{j}{\bar{\sigma }}_{j}=\mu_{j}\sigma$. Then, we have $z_{j}\sim N(0,{\bar{\sigma }}_{j}^{2})$ since *u*_*j*_ ~ *N*(0,1), where *j* = 1,…, *n*. Therefore, $\bar{x}=x+u⊗\bar{\sigma }=x+z.$
Given **S** = ∏_*j*=1_^*n*^[−*b*_*j*_, *b*_*j*_], **S** ^*∗*^(*ɛ*)⊆**S**. Let $\tilde{S}=\{z|z=\bar{x}-x,\bar{x}\in S^{*}(ɛ),x\in S\}$. According to the property of measure, $m(S^{*}(ɛ))=m(\tilde{S})$. One has $P\{\bar{x}\in S^{*}(ɛ)\}=P\{z\in \tilde{S}\}=\int \cdots \int_{\tilde{S}}\prod_{j=1}^{n}(1/\sqrt{2\pi {\bar{\sigma }}_{j}})exp\{-z_{j}^{2}/2{\bar{\sigma }}_{j}^{2}\}dz_{1}\cdots dz_{n}$. Note that $\bar{x},x\in S$ and $z=\bar{x}-x$, so we have $|z_{j}|=|{\bar{x}}_{j}-x_{j}|\leq 2b_{j}=2b$ (*j* = 1,…, *n*), which leads to the following inequality:

(A.2) ∫⋯∫S~∏j=1n12πσ¯jexp⁡−zj22σ¯j2dz1⋯dzn≥∫⋯∫S~∏j=1n12πσ¯jexp⁡−2bj22σ¯j2dz1⋯dzn=mS~∏j=1n12πσexp⁡−2bj2σ2=mS~12πn∏j=1n1σexp⁡−∑j=1n2b2σ2.

That is, we obtain a lower bound of $P\{\bar{x}\in S^{*}\}$ as follows:

(A.3) Px¯∈S∗ɛ≥mS~12πn∏j=1n1σexp⁡−∑j=1n2b2σ2.

Noting that $m(S^{*}(ɛ))=m(\tilde{S})$, we also have $P\{\bar{x}\in S^{*}(ɛ)\}\geq m(S^{*}(ɛ)){\left(1/\sqrt{2\pi }\right)}^{n}(\prod_{j=1}^{n}\left(1/\sigma \right))exp\{-\sum_{j=1}^{n}\left(2b^{2}/\sigma^{2}\right)\}$. (2) Let $f(\bar{\sigma })=\log\left(\prod_{j=1}^{n}\left(1/\sigma \right)\exp\left\{-\sum_{j=1}^{n}\left(2b^{2}/\sigma^{2}\right)\right\}\right)=-\sum_{j=1}^{n}\left(\log\sigma +a_{j}(1/\sigma^{2})\right)$ where *a*_*j*_ = 2*b* ^2^. One has $\max f(\bar{\sigma })\Leftrightarrow \min\sum_{j=1}^{n}\left(\log\sigma +a_{j}(1/\sigma^{2})\right)$, and $d(\log\sigma +a_{j}(1/\sigma^{2}))/d\sigma =0\Rightarrow 1/\sigma -2a_{j}(1/\sigma^{3})=0\Rightarrow \sigma =\sqrt{2a_{j}}$. Noticing that *a*_*j*_ = 2*b*_*j*_^2^ = 2*b* ^2^, we have *σ* = 2*b*, which implies the lower bound of $P\{\bar{x}\in S^{*}(ɛ)\}$ can be improved into

(A.4) mS∗ɛ12πn∏j=1n12bexp−∑j=1n2b22b2=mS∗ɛ12πne−n/2∏j=1n12b=mS∗ɛ12πne−n/22b−n=mS∗ɛ4eπb−n.

Therefore, $P\{{\bar{x}}_{i}^{t}\in S^{*}(ɛ)\}\geq m(S^{*}(ɛ)){\left(4\sqrt{e}\pi b\right)}^{-n}$. (3) By the property of *Ω*_EP_^*∗*^(*ɛ*) and *m*(**S** ^*∗*^(*ɛ*)) in Definition 5, ∀*ɛ* > 0 and *t* = 1,2,…, $P\{\xi_{t}^{C}\in \Omega_{EP}^{*}(ɛ)|\xi_{t-1}^{C}\notin \Omega_{EP}^{*}(ɛ)\}=1-\prod_{i=1}^{k}(1-P\{{\bar{x}}_{i}\in S^{*}(ɛ)\})$. Thus, $P\{\xi_{t}^{C}\in \Omega_{EP}^{*}(ɛ)|\xi_{t-1}^{C}\notin \Omega_{EP}^{*}(ɛ)\}\geq 1-(1-m{\left(S^{*}){\left(4\sqrt{e}\pi b\right)}^{-n}\right)}^{k}\;\;\forall ɛ>0$.

Proof of Corollary 15. (1) Based on the total probability equation, we have

(A.5) λtC=1−λt−1CPξtC∈ΩEP∗ɛ ∣ ξt−1C∉ΩEP∗ɛ+λt−1CPξtC∈ΩEP∗ɛ ∣ ξt−1C∈ΩEP∗ɛ.

Because {*ξ*_*t*_^*C*^}_*t*=0_^+*∞*^ can be considered as an absorbing Markov chain following Lemma 9, *P*{*ξ*_*t*_^*C*^ ∈ *Ω*_EP_^*∗*^(*ɛ*)∣*ξ*_*t*−1_^*C*^ ∈ *Ω*_EP_^*∗*^(*ɛ*)} = 1. Also, *λ*_*t*_^*C*^ = (1 − *λ*_*t*−1_^*C*^)*P*{*ξ*_*t*_^*C*^ ∈ *Ω*_EP_^*∗*^(*ɛ*)∣*ξ*_*t*−1_^*C*^ ∉ *Ω*_EP_^*∗*^(*ɛ*)} + *λ*_*t*−1_^*C*^, and we have 1 − *λ*_*t*_^*C*EP^ = (1 − *λ*_*t*−1_^*C*^)(1 − *P*{*ξ*_*t*_^*C*^ ∈ *Ω*_EP_^*∗*^(*ɛ*)∣*ξ*_*t*−1_^*C*^ ∉ *Ω*_EP_^*∗*^(*ɛ*)}). According to Theorem 14,

(A.6) PξtC∈ΩEP∗ɛ ∣ ξt−1C∉ΩEP∗ɛ≥1−1−mS∗ɛ14eπbnk.

For $m(S^{*}(ɛ)){\left(1/4\sqrt{e}\pi b\right)}^{n}>0$, $d=1-(1-m(S^{*}{\left(ɛ)){\left(1/4\sqrt{e}\pi b\right)}^{n}\right)}^{k}>0$ and lim_*t*→+*∞*_⁡(1 − *d*)^*t*^ = 0. Thus, 1 − *λ*_*t*_^*C*^ ≤ (1 − *d*)(1 − *λ*_*t*−1_^*C*^) = (1 − *λ*_0_^*C*^)(1 − *d*)^*t*^, so lim_*t*→+*∞*_⁡*λ*_*t*_^*C*^ ≥ 1 − (1 − *λ*_0_^*C*^)lim_*t*→+*∞*_⁡(1 − *d*)^*t*^ = 1. Since *λ*_*t*_^*C*^ ≤ 1, lim_*t*→+*∞*_⁡*λ*_*t*_^*C*^ = 1. (2) Given $\alpha =1-(1-m{\left(S^{*}(ɛ)){\left(1/4\sqrt{e}\pi b\right)}^{n}\right)}^{k}>0$, we know that *P*{*ξ*_*t*_^*C*^ ∈ *Ω*_EP_^*∗*^(*ɛ*)∣*ξ*_*t*−1_^*C*^ ∉ *Ω*_EP_^*∗*^(*ɛ*)} ≥ *α* based on Theorem 14. According to Corollary 15, ∀*ɛ* > 0, $E\mu_{C}\leq (1-\lambda_{0}^{C})(1-(1-m(S^{*}(ɛ)){{{\left(1/4\sqrt{e}\pi b\right)}^{n})}^{k})}^{-1}$.

Proof of Theorem 16. (1) For fixed individual $\left(x,\bar{\sigma }\right)$, $\bar{x}=x+\delta {⊗}_{}\bar{\sigma }$, where the stochastic disturbance **δ** follows *n*-dimensional standard Cauchy distribution. Let $z=\delta {⊗}_{}\bar{\sigma }$; that is, *z*_*j*_ = *σδ*_*j*_ (*j* = 1,…, *n*). Then, we have $\bar{x}=x+z$ and *z*_*j*_ ~ (1/*σ*)*C*_*ϕ*=1_(*y*/*σ*) since *δ*_*j*_ ~ *C*_*ϕ*=1_(*y*) (*j* = 1,…, *n*). In a manner similar to the proof of Theorem 14, we derive

(A.7) Px¯∈S∗ɛ=Pz∈S~=∫⋯∫S~1σπn1∏j=1n1+zj2/σ2dz1⋯dzn=∫⋯∫S~1σπn1∏j=1n1+zj2/σ2dz1⋯dzn≥∫⋯∫S~1σπn1∏j=1n1+4b2/σ2dz1⋯dzn=1σπnmS∗ɛ1+4b2/σ2n=mS∗ɛπnσ+4b2σ−n.

(Note that $|z_{j}|=|{\bar{x}}_{j}-x_{j}|\leq 2b$ (*j* = 1,…, *n*).) (2) Letting $f(\bar{\sigma })=-\log\left(\sigma +4b^{2}/\sigma \right)$, we have

(A.8) max⁡fσ¯min⁡σ+4b2σ,dσ+4b2/σdσ0⟹1−4b2σ2=0⟹σ=2b.

It can be improved into $P\{\bar{x}\in S^{*}\}\geq m(S^{*}(ɛ))/{\left(4b\pi \right)}^{n}$ when *σ* = 2*b*. (3) Hence, ∀*ɛ* > 0 and *t* = 1,2,…, if *σ* = 2*b*, *P*{*ξ*_*t*_^*F*^ ∉ *Ω*_EP_^*∗*^(*ɛ*)∣*ξ*_*t*−1_^*F*^ ∈ *Ω*_EP_^*∗*^(*ɛ*)} ≥ 1 − (1 − *m*(**S** ^*∗*^(*ɛ*))/(4*bπ*)^*n*^)^*k*^.

Proof of Corollary 17. (1) According to Theorem 16, *P*{*ξ*_*t*_^*F*^ ∉ *Ω*_EP_^*∗*^(*ɛ*)∣*ξ*_*t*−1_^*F*^ ∈ *Ω*_EP_^*∗*^(*ɛ*)} ≥ 1 − (1 − *m*(**S** ^*∗*^(*ɛ*))/(4*bπ*)^*n*^)^*k*^. 0 < *m*(**S** ^*∗*^(*ɛ*))/(4*bπ*)^*n*^ < 1 due to the second assumption in Section 2.2. Let *θ* = 1 − (1 − *m*(**S** ^*∗*^(*ɛ*))/(4*bπ*)^*n*^)^*k*^ for *t* = 1,2,…. Then 0 < *θ* < 1 and lim_*t*→+*∞*_⁡(1 − *θ*)^*t*^ = 0. Thus, lim_*t*→+*∞*_⁡*λ*_*t*_^*F*^ ≥ 1 − (1 − *λ*_0_^*F*^)lim_*t*→+*∞*_⁡(1 − *θ*)^*t*^ = 1. Also, lim_*t*→+*∞*_⁡*λ*_*t*_^*F*^ = 1 since *λ*_*t*_^*F*^ ≤ 1. (2) Since lim_*t*→+*∞*_⁡*λ*_*t*_^*F*^ = 1, following Corollary 13 and Theorem 16, we find that

(A.9) $$\begin{array}{c} E\mu_{F}\leq \frac{\left(1-\lambda_{0}^{F}\right)}{\left(1-{\left(1-m\left(S^{*}\left(ɛ\right)\right)/{\left(4b\pi \right)}^{n}\right)}^{k}\right)},\;\forall ɛ>0. \end{array}$$
